# Inorganic Pyrophosphate Plasma Levels Are Decreased in Pseudoxanthoma Elasticum Patients and Heterozygous Carriers but Do Not Correlate with the Genotype or Phenotype

**DOI:** 10.3390/jcm12051893

**Published:** 2023-02-27

**Authors:** Matthias Van Gils, Justin Depauw, Paul J. Coucke, Shari Aerts, Shana Verschuere, Lukas Nollet, Olivier M. Vanakker

**Affiliations:** 1 Center for Medical Genetics, Ghent University Hospital, Corneel Heymanslaan 10, 9000 Ghent, Belgium; 2 Department of Biomolecular Medicine, Ghent University, Corneel Heymanslaan 10, 9000 Ghent, Belgium; 3 Ghent Ectopic Mineralization Research Group, 9000 Ghent, Belgium

**Keywords:** *ABCC6*, heterozygous carriers, inorganic pyrophosphate, pseudoxanthoma elasticum, genotype–phenotype correlation, biomarker

## Abstract

Pseudoxanthoma elasticum (PXE) is a rare ectopic calcification disorder affecting soft connective tissues that is caused by biallelic *ABCC6* mutations. While the underlying pathomechanisms are incompletely understood, reduced circulatory levels of inorganic pyrophosphate (PPi)—a potent mineralization inhibitor—have been reported in PXE patients and were suggested to be useful as a disease biomarker. In this study, we explored the relation between PPi, the *ABCC6* genotype and the PXE phenotype. For this, we optimized and validated a PPi measurement protocol with internal calibration that can be used in a clinical setting. An analysis of 78 PXE patients, 69 heterozygous carriers and 14 control samples revealed significant differences in the measured PPi levels between all three cohorts, although there was overlap between all groups. PXE patients had a ±50% reduction in PPi levels compared to controls. Similarly, we found a ±28% reduction in carriers. PPi levels were found to correlate with age in PXE patients and carriers, independent of the *ABCC6* genotype. No correlations were found between PPi levels and the Phenodex scores. Our results suggest that other factors besides PPi are at play in ectopic mineralization, which limits the use of PPi as a predictive biomarker for severity and disease progression.

## 1. Introduction 

Ectopic mineralization—the pathologic deposition of calcium salts in soft tissues—is a complex process underlying a heterogenous group of disorders—both common (such as atherosclerosis, kidney disease and stroke) and orphan diseases—and is associated with significant morbidity and mortality. Evidence has emerged in recent years that ectopic mineralization arises out of a dynamic deregulation of several gene regulatory networks, proteins and metabolic alterations reflecting complex perturbations. A central mediator in these processes is inorganic pyrophosphate (PPi), a potent calcification inhibitor that acts by binding and coating crystal nucleation sites, thus halting ectopic mineralization formation [1]. PPi is produced by the conversion of extracellular ATP into adenosine monophosphate (AMP) and PPi by the ectonucleotidase enzyme ENPP1 [2]. Circulating PPi has a short half-life of ±30 min, in part due to hydrolyzation into pro-mineralizing phosphate (Pi) molecules by alkaline phosphatases (ALPL). In turn, ALPL activity is inhibited through adenosine release from AMP by CD73 [3].

Another important protein in the homeostasis of PPi was recently identified as *ABCC6*, an adenosine triphosphate (ATP)-binding transporter protein encoded by the *ABCC6* gene. Biallelic pathogenic variants in *ABCC6* are known to cause pseudoxanthoma elasticum (PXE), an autosomal recessive disorder in which the calcification and fragmentation of elastic fibers results in skin (papular lesions and increased skin laxity), eye (angioid streaks, choroidal neovascularization and hemorrhage) and cardiovascular symptoms (peripheral artery disease and stroke), though the severity is highly variable between patients [4,5,6]. In PXE families, heterozygous carriers of one *ABCC6* pathogenic variant can present a partial PXE phenotype. While they do not develop skin lesions and PXE retinopathy is rare, they can present calcifications in the retina and abdominal organs and have an increased risk for cardio- and cerebrovascular complications [7,8,9,10,11].

Because of the predominant expression of *ABCC6* in liver and kidney, PXE is considered a metabolic disorder driven by the absence of systemic substrates provided from the liver through *ABCC6*. The *ABCC6* substrate remains elusive, but *ABCC6*—at least indirectly—regulates the release of ATP from hepatocytes into the circulation [12,13], thereby contributing to PPi production and mineralization homeostasis [3]. While *ABCC6*-mediated ATP release appears to be the main source of PPi, extracellular ATP may also be released by ANKH [14].

Decreased plasma levels of PPi are an important contributor to the ectopic mineralization in PXE. Significantly decreased PPi levels have been reported in PXE patients and *Abcc6^−/−^* rodent models [12,13,15]. Moreover, PPi deficiency is critical to the development of generalized arterial calcification of infancy (GACI), an ectopic calcification disorder with genetic and clinical overlap with PXE [16,17]. Furthermore, differential expressions and/or activities of ANKH, ALPLs and CD73 have been reported in PXE [13,15,18,19,20], leading to the consensus that a pro-mineralizing shift in the PPi/Pi balance is a significant driver of the PXE calcification phenotype. While many other factors can further negatively affect the mineralization homeostasis in PXE [3,21,22], recent studies using PPi supplementation in *Abcc6^−/−^* mice suggest that increasing PPi levels can help in preventing the progression of mineralization [23,24]. In this study, we demonstrated that PPi plasma levels are not only decreased in PXE patients but also in heterozygous carriers and may thus contribute to their partial phenotype. However, we could not find any correlation with the severity of the phenotype or with the *ABCC6* genotype. Together with the observed overlap in PPi levels between patients, carriers and controls, this convinced us that PPi plasma levels are not a reliable biomarker for PXE.

## 2. Materials and Methods

### 2.1. Molecular and Clinical Evaluation

For all patients, heterozygous carriers and controls, a molecular analysis of the *ABCC6* gene was performed as previously described [11,25]. All *ABCC6* variants were classified using the Sherloc classification of pathogenicity [26,27]. All patients had a histological diagnosis of PXE, and the disease severity was assessed using the PXE International Phenodex scoring system at the time of blood sampling for PPi measurement [28]. Scoring was performed for skin (S0–S3), eye (E0–E4), vasculature (V0–V3), cardiac (C0–C2) and gastrointestinal (G0–G1) symptoms. Data on putative confounders (i.e., smoking, hypertension, hypercholesterolemia, diabetes and obesity) were collected. Informed consent was obtained from all participants, and the tenets of the Declaration of Helsinki were followed. This study was approved by the ethical committee of the Ghent University Hospital.

### 2.2. Plasma PPi Analysis

Fasting blood samples were collected from PXE patients, carriers and controls using 4 citrate BD-Vacutainers (363,083, BD) per sampling and stored on ice. The samples were immediately centrifuged at 1000× *g* for 10 min at 4 °C. Patient plasma was pooled, redistributed to Centrisart I MWCO 300,000 Da tubes (Item No. 13,279—E, Sartorius, Göttingen, Germany) and centrifuged at 2300× *g* for 30 min at 4 °C. Purified plasma was then collected, anonymized and stored at −80 °C.

The plasma PPi content was determined using a two-step ATPase luminescence assay prepared on ice. For each plasma sample, 250 µL of ATP conversion mix (825 mU of ATP sulfurylase (M0394L, Bioké NV, Leiden, The Netherlands), 64 µM APS (SC-214506, Bio-Connect, Huissen, The Netherlands), 31 mM HEPES (pH = 7.4) and 104 µM Mg_2_Cl) was prepared. To 58 µL of plasma, 2 µL of 0 µM ATP and 20 µL of ATP conversion mix were added. PPi was enzymatically converted to ATP in a PCR machine (30 min at 37 °C and 10 min at 90 °C). To generate an internal calibration curve, 11 aliquots of 58 µL plasma samples were spiked with 2 µL of 0–50 µM ATP (in increments of 5 µM) and 20 µL of heat-inactivated ATP conversion mix.

**Figure 1:**
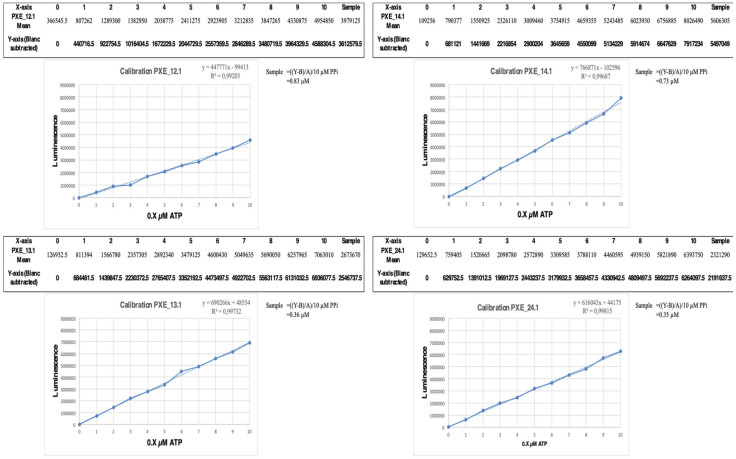
Determination of PPi plasma content for the 4 samples. Means for all duplicates (shown individually in Supplementary Table S1) were determined, and blanks were subtracted. These mean values were plotted, and the linear gradient was calculated. Conversion of the formulae (including a 10× correction) yielded the PPi content (µM) of each sample. The gradient and intercept varied between patient samples, again suggesting that the plasma content may affect the luminescence assay readout. For instance, sample 12.1 had a higher PPi level at readout compared to the other samples, notwithstanding its lower luminescence output.

The 40 µL sample and calibration curve aliquots were distributed in pairs on a 96-well plate, and 10 µL of Bactiter-Glo was added to each well. After mixing for 2 min, the luminescence was measured using a Glomax (E7031, Promega, Leiden, The Netherlands). Linear calibration curves were established, and plasma PPi values were derived from the first-degree equations (Figure 1 and Supplementary Table S1).

### 2.3. Statistical Analysis

A statistical analysis was performed via SPSS26 software (IBM, Chicago, IL, USA). The sample distribution was determined via Kolmogorov–Smirnov testing for normality, and subsequent analyses via (independent) *t*-testing, (repeated measures) ANOVA, chi-square (nominal variables) or their non-parametric counterparts were performed. The correlations between variables were first estimated using Pearson or Spearman analyses, depending on the variable distribution. Regression analyses were subsequently performed to estimate the predictive power of variables on particular dependents. The results were significant at *p* ≤ 0.05.

## 3. Results

### 3.1. Demographic, Molecular and Clinical Characteristics of the Cohorts

Samples from 78 Caucasian PXE probands, 69 carriers and 14 controls were analyzed. Table 1 shows the demographic characteristics of the patients, heterozygous carriers and the control cohort. Sex and age were normally distributed; no significant differences were found for these characteristics between the groups (*p* > 0.05). The molecular and clinical characteristics of the patient cohort are shown in Supplementary Table S2. In all but three patients, biallelic *ABCC6* variants were identified. In total, 126 variants were pathogenic (class 5), 8 were likely pathogenic (class 4) and 19 were variants of unknown significance (class 3), with 12 and 1 of these identified as likely pathogenic (C3LP) and likely benign (C3LB), respectively. The characteristics of the carriers and controls are shown in Supplementary Table S3. In 44 and 6 carriers, respectively, a heterozygous *ABCC6* pathogenic (class 5) or likely pathogenic (class 4) variant was found. In total, 19 carriers had a class 3 variant, of which 12 were C3LP variants.

The effects of other confounding factors in our patient cohort were investigated. Notably, 8 patients were smokers; one patient had type II diabetes mellitus; no patients had untreated hypertension, but 5 patients were taking anti-hypertensive drugs and 3 patients and 23 patients had untreated and medication-controlled hypercholesterolemia, respectively. However, these confounders did not correlate with sex, age or the measured plasma PPi levels (*p* > 0.05) and were therefore considered non-informative (Table 2).

The effects of putative confounding factors, i.e., smoking (yes/no), diabetes mellitus (no, type I or type II), hypertension (yes/no) and hypercholesterolemia (untreated/no or treated), were analyzed according to sex (female/male), PPi levels (µM) and age (years). Diabetes mellitus was diagnosed according to the WHO criteria (a fasting blood sugar level of 126 mg/dL, a 2 h oral glucose tolerance test result of 200 mg/dL and hemoglobin A1c of 6.5% or higher). Hypertension was defined as a systolic and/or diastolic blood pressure higher than 140/90 mmHg. Hypercholesterolemia was defined as a fasting low-density lipoprotein cholesterol (LDL-C) concentration > 115 mg/dL. No significant effects were identified (all *p* > 0.05.).

### 3.2. PPi Levels Were Reduced in Patients and Carriers Compared to Controls, Though Overlap between Biological Ranges Was Observed

**Figure 2:**
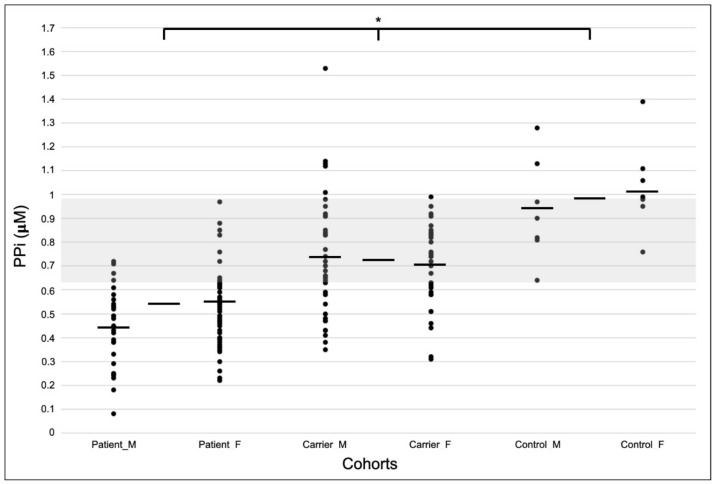
Distributions of measured plasma PPi values per cohort and sex. Samples were plotted according to cohort (PXE patients, carriers and controls) and sex (male and female). Horizontal bars represent mean values (µM), and the bars in between represent the cohort means. For exact values, see Table 1. The distributions indicate some overlap between cohorts in the 0.64–0.98 µM range (i.e., the lowest control to the highest PXE value, respectively (gray bar)). Significant mean differences in PPi were found between all patient, heterozygous carrier and control cohorts (* *p* < 0.001).

The distribution of the measured PPi values was normal for each cohort (*p* > 0.05), and the spread per cohort and sex is shown in Figure 2. While some overlap in PPi values was apparent, significant mean differences between all three cohorts were found (*p* < 0.001). Table 2 summarizes the measured plasma PPi levels (means ± SDs (µM)) of the PXE patients, heterozygous carriers and the control cohorts and per sex. The relative comparison between the cohorts—with the control cohort as a baseline—showed a significant mean reduction of ±50% in PPi levels in the PXE patients. Similarly, a significant ±28% reduction was observed for carriers.

The PXE patient, heterozygous carrier and control sample cohorts are displayed according to sex (male/female) and as a group (total). The N represents the number of samples, age is mean ± SD (years) and PPi is mean ± SD (µM). Significant differences in PPi levels were found between the three cohorts (ANOVA after Bonferroni correction: PXE–heterozygous carrier, *p* < 0.00001; PXE–control, *p* < 0.00001; heterozygous carrier–control, *p* = 0.000083).

**Figure 3:**
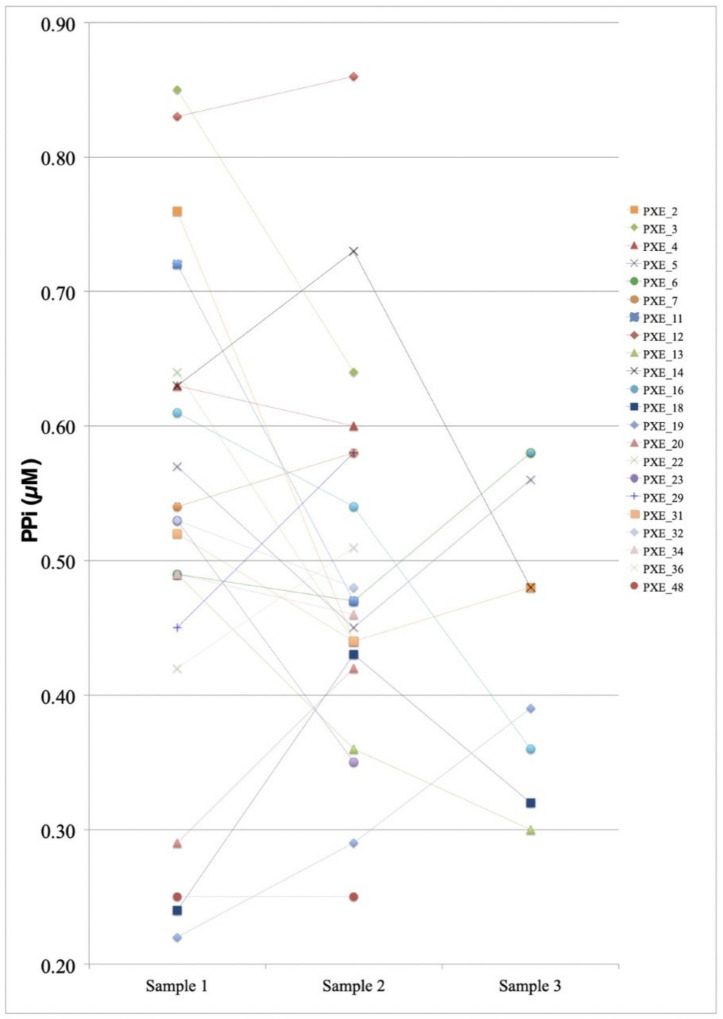
Variance in measured PPi levels between patients with multiple samples. Multiple blood samples were obtained from 22 PXE patients on a yearly basis. For 14 patients, 2 samples were obtained, and for 8 patients 3 samples were obtained. Comparison between measured samples revealed no significant plasma PPi variation between samples (*p* > 0.05, *t*-test and ANOVA). While some repeat-measurement PPi values appeared relatively stable (e.g., PXE_5, PXE_6 and PXE_48), others fluctuated more strongly (e.g., PXE_14).

Moreover, we looked at the fluctuation in PPi levels between yearly repeated samples in 14 PXE patients with two samples and 8 PXE patients with three samples (Supplementary Table S2). When comparing the three groups (sample 1: N = 22, sample 2: N = 22 and sample 3: N = 8; Figure 3), we noted that some patients appeared to have fluctuations in the measured plasma PPi levels, but overall no significant differences were found between the compared groups (repeated-measures ANOVA (N = 8/8/8): F = 0.736, *p* > 0.05. Paired-samples *t*-test: samples 1–2: (N = 22/22), *p* > 0.05; samples 2–3 (N = 8/8), *p* = 0.527; samples 1–3: (N = 8/8), *p* > 0.05).

### 3.3. Plasma PPi Levels Correlated with Age in PXE but Not with Sex

Next, we investigated whether sex or age were correlated with the measured PPi levels. First, we analyzed sex-based differences in each cohort, but no significant differences between men and women were found in patients, carriers or controls (*p* > 0.05; Table 2).

**Figure 4:**
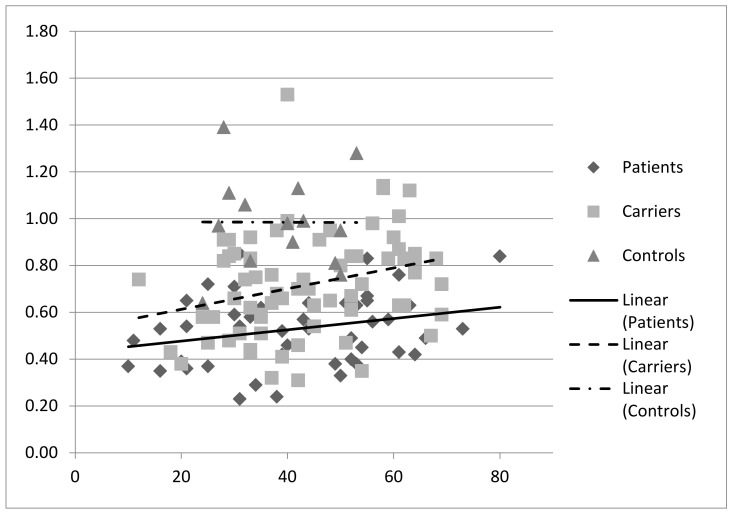
Correlation analysis of PPi and age, indicating a significant positive correlation of the PPi plasma levels with age in the PXE and heterozygous carrier cohorts (*p* < 0.05) but not in the controls.

Correlation analyses suggested that in patients and carriers PPi levels significantly increased with age (*p* < 0.05; Figure 4), but no such effect was observed in controls (*p* > 0.05; Figure 4).

### 3.4. Plasma PPi Levels Did Not Correlate with the ABCC6 Genotype

To determine the correlations between the plasma PPi levels and the patient genotypes, the *ABCC6* variants of the patient cohort were classified according to the Sherloc variant classification (Supplementary Table S2) [27]. We then categorized the patient cohort into six genotype groups, depending on the expected effect of each variant, i.e., variants likely resulting in erroneous mRNA products (deletions/frameshifts/splice site variants (D)), variants resulting in truncated/unstable proteins (nonsense (N)) and variants likely affecting protein function (missense (M)). Three patients with a single *ABCC6* pathogenic variant were excluded from the analyses, resulting in 75 patients for a univariate analysis with correction for sex and age (Table 3).

Stepwise analyses were performed with increased stringency for genotype inclusion: all C5-C3 variants (*n* = 75), C5-C3LP (likely pathogenic, excluding likely benign and unknown C3 variants; *n* = 71), C5-C4 (*n* = 40) and only C5 (*n* = 39). None of the generated models could explain the measured PPi levels.

Comparable analyses for heterozygous carrier genotypes were performed without significant results (*p* > 0.05). Thus, we did not identify genotype–PPi correlations.

### 3.5. Plasma PPi Levels Did Not Correlate with the Phenodex Scores

Regression analyses (Table 4) were performed on the Phenodex scores with respect to PPi, age and sex. For skin lesion severity, sex (i.e., being female; *p* = 0.045) was associated with higher scores. Ocular severity was only significantly predicted by age (*p* < 0.001).

Given the low incidences of cardiovascular events in our cohort (V1: *n* = 9, V2: *n* = 4, V3: *n* = 3, C1: *n* = 2 and C2: *n* = 2), we first opted for a binary logistic regression (i.e., the absence/presence of a lesion). For the vascular outcome (absent: *n* = 64 and present: *n* = 14), sex was found to be non-informative, while age (but not PPi) significantly and inversely contributed (*p* < 0.01). For the cardiac outcome (absent: *n* = 75 and present: *n* = 3) sex, age and PPi were non-informative (*p* > 0.05). When performing ordinal logistic regressions, the severity of vascular lesions was only predicted by age (*p* < 0.01). As with the binary analysis, PPi and age did not contribute significantly to the cardiac Phenodex model (*p* > 0.05).

## 4. Discussion and Conclusions

Since the initial reports that inorganic pyrophosphate is reduced, PPi has been demonstrated to play a pivotal role in the occurrence of ectopic mineralization in PXE [12,29]. Until recently, the human data published on the relation between *ABCC6* and PPi had been at a relatively small scale due to the rarity of the disorder [15,19,29], and data on PPi levels in heterozygous carriers were not available.

We observed a ±50% reduction in circulatory plasma PPi levels in our PXE patient cohort relative to healthy controls. Heterozygous carriers had a ±28% relative reduction in PPi levels between patients and controls. Despite some overlap, the mean PPi levels of all cohorts were significantly different. Thus, in general, PPi levels are directly dependent on the normal expression of both *ABCC6* alleles.

The patient PPi levels in our study are in line with earlier reports. Jansen et al. reported a relative reduction in plasma PPi of ±60% using CTAD-EDTA tubes [29]. Similarly, Sanchez-Tévar et al. noted significant relative reductions of ±43% (0.35 ± 0.15 µM) and ±22% (1.11 ± 0.26 µM) when measuring CTAD- and EDTA-based PPi samples of the same patients, respectively [15]. Lefthériotis et al. noted a similar 50% reduction [19]. As we used a citrate-based assay, such differences in reduction—both relative and absolute—indicate that the anticoagulant may significantly impact the PPi signal readout, as exemplified by the difference found between CTAD and EDTA by Sanchez-Tévar et al. [15]. Such an effect of the anticoagulant was recently illustrated by Lundkvist et al., who showed that the amount of PPi detected depended on the anticoagulant used in the unfiltered plasma, with CTAD and heparin yielding higher concentrations compared to EDTA [30]. Moreover, we performed an internal calibration and noted that the readout values could vary between different samples despite having been spiked with equal amounts of ATP, indicating that unknown factors in the plasma can affect the readout and may be responsible for part of the variation between different studies (Supplementary Table S1). The value of an internal ATP standard to obtain reliable measurement results was also recently shown by Lundkvist et al. [30]. We cannot exclude that ethnic factors also affect PPi levels [31,32,33,34,35,36]. Though it remains a matter of debate which methodology measures the true and exact absolute PPi plasma levels, if any, the different available methods seem to be reliable to document the relative decreases in PPi plasma levels in PXE compared to controls. Indeed, our study reaffirmed that PPi is significantly reduced in PXE, though it is quite variable between patients. We observed in several PXE patients—in particular females—that their PPi levels were comparable to heterozygous carrier or control levels. This confirms that, at least in some patients, mechanisms independent of PPi must also play a role in PXE. A similar observation was made in *Abcc6^−/−^* mice in which PPi levels were increased by the global overexpression of ENPP1. Although plasma PPi was increased to the levels found in wild-type mice, the transgenic animals still suffered from small mineralization foci [37].

Mineralization in PXE develops progressively over time [38]. Moreover, tissue calcification, in particular vascular calcification, is a hallmark of aging [39,40]. We thus questioned if PPi levels were affected by age and found significant positive correlations between PPi levels and age in PXE patients and carriers but not in controls. The correlation with age in PXE patients was also noted by Lefthériotis et al., though this was only present in female patients [19]. This was in contrast to the PXE murine model, where no correlation between age and the quantified purinergic metabolites in the plasma of *Abcc6^−/−^* and wild-type mice was found, which could have been due to species-specific differences or the small number of analyzed samples [13]. We currently cannot explain the increase in PPi with age in PXE patients and heterozygous carriers, and it remains to be seen whether this correlation can be confirmed in larger independent cohorts.

Besides the variable distribution, the plasma PPi levels did not correlate significantly with Phenodex score severity, similar to the findings of Lefthérotis et al. for cumulative Phenodex scores [19]. This indicates that a snapshot of the plasma PPi is not a reliable marker for the risk stratification of patients, nor does it seem to be a good surrogate end point to be used in clinical trials. It could be that the extracellular tissue content of PPi correlates more to disease severity than the circulatory levels. However, no reliable methods are currently available to measure tissue PPi content. Further, it is already known that PPi is only one of the many factors involved in the calcification process in PXE. Several other pathophysiological mediators and mechanisms—Fetuin-A, MGP, alkaline phosphatase, magnesium and DNA damage response activation—have been described as contributing to the ectopic calcification in PXE and may be explanations for why no significant correlations can be found with the PXE phenotype when the correlation analysis focuses only on one mediator [41,42,43,44,45,46]. Though recently a significant correlation was found with the calcification propensity time (T50), the results of this pilot study await further confirmation in larger independent study cohorts [45]. It may be that a good correlation with the complex PXE phenotype can only be achieved using a multivariable score, taking into account all of the abovementioned mediators. Finally, it cannot be fully excluded that if PPi plasma levels are followed prospectively over time a correlation with the disease progression may be present. This may, however, be challenging to document in view of the slowly progressive nature of the PXE phenotype and the important interfamilial variability. For this, a validated clinical biomarker would be an important asset.

Because of the relatively broad range of PPi plasma levels in patients and carriers, we wondered if the nature of the *ABCC6* variants would impact the PPi levels. However, we could not detect any effect of the *ABCC6* genotype. Clearly, the regulation of circulatory PPi is much more complex than just *ABCC6* activity and involves other (epi)genomic and environmental factors [3,47].

In conclusion, we confirmed decreased PPi levels in PXE patients and heterozygous carriers in an independent cohort, though in several patients the PPi levels were within the heterozygous carrier or control range. This suggests that other pathophysiological factors are at play in at least some patients, which limits the use of PPi as a diagnostic marker and as a predictive biomarker for disease severity and progression. Our results should be evaluated in other independent cohorts of different ethnicities and genetic backgrounds. Moreover, in common disorders such as chronic kidney disease and diabetes mellitus, in which vascular calcification is correlated with increased risk of cardiovascular complications, the predictive value of PPi plasma levels for these adverse events should be evaluated [48]. To this end, the measurement protocol that we have optimized can be used in clinical routines, as it has a turnaround time of 4 h from sampling to result. Further, samples can be temporarily stored at −80° after processing without affecting the measurement results, making batch analysis possible.

The limitations of this study include that it is a cross-sectional study and that we did not observe a correlation with the mineralization load. However, we found that the Phenodex scoring grasped the actual symptoms of the patients—particularly for the ocular and cardiovascular features—better than a calcification score and considered it more appropriate to evaluate whether PPi levels could be used as a clinical biomarker. For the genotype correlations, a limitation is the limited available knowledge on the effect of *ABCC6* missense variants. These variants were all considered together, though we cannot exclude that their functional effects may be different. Further, there is currently limited knowledge on the effects of the variants that are classified as class 3 variants of unknown significance, and therefore we cannot be completely sure about their implication in PXE [27]. We took this limitation into account by also performing a correlation analysis with just the class 5 and/or class 4 variants.

## Figures and Tables

**Table 1:** Overview of demographics and PPi levels of the study cohorts.

Cohort	N	Age (Years)	PPi (µM)	Relative PPi Level (%)
Male PXE	28	39.21 ± 15.52	0.441 ± 0.163	47.1
Female PXE	50	44.12 ± 15.99	0.565 ± 0.112	54.6
PXE Total	78	42.35 ± 15.90	0.497 ± 0.092	50.4
Male Carriers	37	44.32 ± 14.31	0.731 ± 0.253	78.1
Female Carriers	32	43.41 ± 14.10	0.703 ± 0.174	67.9
Carriers Total	69	43.90 ± 14.12	0.710 ± 0.220	72.1
Male Controls	7	38.43 ± 10.89	0.936 ± 0.214	95.0
Female Controls	7	38.86 ± 09.39	1.034 ± 0.236	105.0
Controls Total	14	38.64 ± 09.77	0.985 ± 0.202	100.0

**Table 2:** Confounder analyses.

Analysis of Additional Confounders (*n* = 78)		Sex			PPi			Age		
		M	F	Test and *p*-Value	N	Mean ± SD (µM)	Test and *p*-Value	N	Mean ± SD (Years)	Test and *p*-Value
Smoking	No	23	47	Chi-square *p* = 0.097	70	0.556 ± 0.09	*t*-Test *p* = 0.211	70	42.37 ± 16.46	*T*-Test *p* = 0.977
	Yes	5	3		8	0.461 ± 0.193		8	42.25 ± 10.51	
Diabetes Mellitus	No	28	49	Chi-square *p* = 0.692	77	0.623 ± 0.158	Mann–Whitney *U*-test *p* = 0.581	77	42.67 ± 16.01	Mann–Whitney *U*-test *p* = 0.129
	Type II	0	1		1	0.585		1	66.00	
Hypertension	No/Treated	28	50	/	78	0.565 ± 0.092	/	78	42.35 ± 15.90	/
	Untreated	0	0		0	/		0	/	
Hypercholesterolemia	No/Treated	26	49	Chi-square *p* = 0.257	75	0.527 ± 0.161	Mann–Whitney *U*-test: *p* = 0.710	75	42.21 ± 16.55	Mann–Whitney *U*-test: *p* = 1.000

**Table 3:** PPi–genotype analyses.

C5-C3 Model: *p* = 0.051, R^2^adj. = 0.166			
	*p*	B or Δ	95% CI
Intercept	<0.001	0.398	0.268/0.671
Age	0.062		
Sex	0.078		
C5-C3 Genotype	0.083		
Sex*C5-C3 Genotype	0.482		
C5-C3LP Model: *p* = 0.117			
C5-C4 Model: *p* = 0.326			
C5 Model: *p* = 0.280			

Univariate analysis of variance testing for PPi–genotype analysis (with age and sex as confounders). All models were non-significant. For the C5-C3 borderline non-significant result, B is shown for continuous variables, Δ indicates the mean difference measured for categorical variables and 95% CI is the 95% confidence interval.

**Table 4:** PPi–Phenodex analyses.

Binary Logistic Regression			
Vascular Phenotype (Absent: *n* = 64, Present: *n* = 14)			
	*p*	B	95% CI
Constant	0.059	−4.313	
Age	0.010	0.111	1.014/1.111
PPi	0.215	−2.301	−4.625/3.798
Cardiac Phenotype (Absent: *n* = 75, Present: *n* = 3)			
Constant	0.149	−5.062	
Age	0.106	0.059	0.987/1.141
PPi	0.301	−3.359	−15.654/20.334
Ordinal Logistic Regression			
Skin Phenodex Scores (S0: *n* = 10, S1: *n* = 12, S2: *n* = 41, S3: *n* = 15)			
	*p*	Estimate or Δ	95% CI
Sex	0.045	1.035	0.059–0.973
Age	0.411	0.048	0.939/1.026
PPi	0.403	2.913	0.003/10.074
Eye Phenodex Scores (E0: *n* = 1, E1: *n* = 4, E2: *n* = 46, E3: *n* = 24, E4: *n* = 3)			
Sex	0.697	−0.738	−1.831/1.852
Age	<0.001	0.156	0.071/0.200
PPi	0.668	−1.925	−5.973/3.378
Vascular Phenodex Scores (V0: *n* = 63, V1: *n* = 9, V2: *n* = 4, V3: *n* = 2)			
Age	0.009	0.116	0.032/0.200
PPi	0.199	−2.546	−15.652/3.643
Cardiac Phenodex Scores (C0: *n* = 74, C1: *n* = 2, C2: *n* = 2)			
Age	0.145	0.568	−0.100/1.132
PPi	0.243	−38.798	−76.528/5.917

Binary and ordinal logistic regressions were performed to determine PPi–Phenodex correlations. Age was a significant positive predictor for the severity of eye and vascular scores. PPi levels were a significant negative predictor of vascular scores. B is shown for continuous variables, Δ indicates the mean difference measured for categorical variables and 95% CI is the 95% confidence interval.

## Data Availability

The data presented in this study are available on request from the corresponding author. The data are not publicly available due to privacy reasons.

## Supplementary Materials

The following supporting information can be downloaded at: https://www.mdpi.com/article/10.3390/jcm12051893/s1. Table S1: Readout data from a 96-well plate (4 samples in duplo). Table S2: Detailed data of the PXE patient cohort. Table S3: Characteristics of the heterozygous carrier and control study cohort.
