# Supplementary material for: Inorganic Pyrophosphate Plasma Levels Are Decreased in Pseudoxanthoma Elasticum Patients and Heterozygous Carriers but Do Not Correlate with the Genotype or Phenotype
Source: J Clin Med. 2023 Feb 27;12(5):1893. doi: 10.3390/jcm12051893 (PMC10003929; doi:10.3390/jcm12051893)
Supplement: Supplementary file 1 [file jcm-12-01893-s001.zip › jcm-2087069-supplementary.pdf]

Supplementary Materials

Supplementary Table S1. Readout data from a 96-well plate (4 samples in duplo).

	Blanc	0.1 µM ATP	0.2 µM ATP	0.3 µM ATP	0.4 µM ATP	0.5 µM ATP	0.6 µM ATP	0.7 µM ATP	0.8 µM ATP	0.9 µM ATP	1.0 µM ATP	Sample
**PXE_ 121**	367445	839820	1313790	1402470	2033380	2409940	2883490	3299450	3897290	4218280	4996480	4384720
	365646	774704	1264810	1363430	2044170	2412610	2964320	3126220	3797240	4443470	4913220	3573530
**PXE_ 131**	128826	851859	1604030	2421180	2938550	3525560	4708420	5153680	5817860	6249730	7123710	2719410
	125039	770929	1529530	2293430	2846130	3432690	4492440	4945590	5562240	6266200	7002310	2627930
**PXE_ 141**	113526	776395	1656100	2298680	3094040	3881020	4750610	5337940	6185960	6890140	8268460	5765080
	104986	804359	1445750	2353540	2924880	3628810	4568100	5149030	5861900	6623630	7784520	5447530
**PXE_ 241**	122479	777198	1537940	2151310	2577880	3350150	3780520	4582420	5092730	6245950	6452210	2478700
	136826	741612	1503390	2046250	2567900	3269020	3795700	4338770	4785570	5397830	6335290	2163880

Example of Glomax luminescence assay (i.e. ATP luciferase assay) readout for 4 PXE samples (12.1, 13.1, 14.1 and 24.1) in duplo; the readouts of the two duplicates for each sample and condition are shown. The top row shows final concentration of ATP added by internal calibration. Readout values are differential between patients despite being spiked with equal amounts of ATP (e.g. at 1.0µM ATP) suggesting that components in the plasma may affect signal output and that individual calibration is recommended.

Supplementary Table S2. Detailed data of the PXE patient cohort.

**N**	**S**	**Age**	**PPi (µM)**			**S**	**E**	**V**	**C**	**G**	**T**	**Variant 1 & Class**		**Variant 2 & Class**		**Cat.**
**1**	M	50	0.33			2	3	0	0	0	5	c.1132C>T; p.(Q378*)	C5	c.1132C>T; p.(Q378*)	C5	N+N
**2**	F	31	0.76	0.44 (32)	0.48 (33)	3	2	0	0	0	5	g.65280_73844del	C5	c.1892_1943+26del78; p.(I631fs*1)	C5	D+D
**3**	F	63	0.85	0.64 (65)		2	3	0	0	0	5	c.3421C>T; p.(R1141*)	C5	c.3907G>C; p.(A1303P)	C5	N+M
**4**	F	43	0.63	0.60 (45)		2	2	0	0	0	4	c.3421C>T; p.(R1141*)	C5	c.1996G>T; p.(G666V)	C5	N+M
**5**	F	16	0.57	0.45 (17)	0.56 (18)	0	1	0	0	0	1	c.2278C>T; p.(R760W)	C5	g. 65280_73844del	C5	D+M
**6**	F	21	0.49	0.47 (22)	0.58 (23)	2	2	0	0	0	4	c.2278C>T; p.(R760W)	C5	g.65280_73844del	C5	D+M
**7**	F	59	0.54	0.58 (61)		2	2	3	0	0	7	c.3421C>T; p.(R1141*)	C5	c.3421C>T; p.(R1141*)	C5	N+N

**8**	F	73	0.57			2	3	0	0	0	5	c.3188T>G; p.(L1063R)	C3LP	c.3188T>G; p.(L1063R)	C3LP	M+M
**9**	F	56	0.35			3	3	2	0	0	8	c.3907G>C; p.(A1303P)	C5	c.3421C>T; p.(R1141*)	C5	N+M
**10**	F	25	0.56			1	2	0	0	0	3	c.3364delT; p.(S1122Lfs*33)	C5	c.3364delT; p.(S1122Lfs*33)	C5	D+D
**11**	M	55	0.72	0.47 (56)		2	3	0	0	0	5	g.65280_73844del	C5	g.65280_73844del	C5	D+D
**12**	F	53	0.83	0.86 (56)		3	3	0	0	0	6	c.3907G>C; p.(A1303P)	C5	c.3421C>T; p.(R1141*)	C5	N+M
**13**	M	43	0.49	0.36 (44)	0.30 (45)	2	3	0	0	0	5	c.4213G>A; p.(G1405S)	C5	c.3074T>C; p.(L1025P)	C3LP	M+M
**14**	F	52	0.63	0.73 (53)	0.48 (54)	2	2	0	0	0	4	c.3421C>T; p.(R1141*)	C5	g.65280_73844del	C5	D+N
**15**	F	61	0.72			3	2	0	0	0	5	c.3421C>T; p.(R1141*)	C5	c.1355C>A; p.(A452D)	C3LP	N+M
**16**	M	38	0.61	0.54 (39)	0.36 (40)	2	3	0	0	0	5	c.3907G>C; p.(A1303P)	C5	g.65280_73844del	C5	D+M
**17**	F	31	0.43			2	3	0	0	0	5	c.3940C>T; p.(R1314W)	C5	c.3940C>T; p.(R1314W)	C5	M+M
**18**	M	34	0.24	0.43 (35)	0.32 (36)	2	2	0	0	0	4	c.3941G>A; p.(R1314Q)	C5	g.65280_73844del	C5	D+M
**19**	F	33	0.22	0.29 (34)	0.39 (35)	2	2	0	0	0	4	c.3941G>A; p.(R1314Q)	C5	g.65280_73844del	C5	D+M
**20**	M	51	0.23			2	3	2	2	0	9	c.3907G>C; p.(A1303P)	C5	c.1636-11_1636-10delinsAG	C3LP	D+M
**21**	M	44	0.29	0.42 (45)		2	2	2	0	0	6	c.3364delT; p.(S1122Lfs*33)	C5	c.1552C>T; p.(R518*)	C5	D+N
**22**	M	21	0.58			2	2	0	0	0	4	c.3506+2T>C	C5	g.5001_78907del	C5	D+D
**23**	F	80	0.64	0.44 (81)		1	3	0	0	0	4	c.1552C>T; p.(R518*)	C5	c.3421C>T; p.(R1141*)	C5	N+N

**24**	F	25	0.53	0.35 (27)	2	2	0	0	0	4	c.3421C>T; p.(R1141*)	C5	c.3188T>G; p.(L1063R)	C3LP	N+M
**25**	F	49	0.65		1	2	0	0	0	3	c.1132C>T; p.(Q378*)	C5	c.1171A>G; p.(R391G)	C3LP	N+M
**26**	F	40	0.34		3	2	0	0	0	5	c.3364delT; p.(S1122Lfs*33)	C5	c.1194C>G; p.(S398R)	C3LP	D+M
**27**	F	55	0.47		2	3	0	0	0	5	c.1552C>T; p.(R518*)	C5	c.3421C>T; p.(R1141*)	C5	N+N
**28**	F	35	0.38		2	2	0	0	0	4	c.3421C>T; p.(R1141*)	C5	c.3437T>C; p.(F1146S)	C3U	N+M
**29**	F	39	0.46		3	2	0	0	0	5	c.3421C>T; p.(R1141*)	C5	c.1996G>T; p.(G666V)	C5	N+M
**30**	F	16	0.45	0.58 (17)	2	1	0	0	0	3	c.3421C>T; p.(R1141*)	C5	g.5001_78907del	C5	D+N
**31**	F	52	0.62		0	3	0	0	0	3	c.2782G>A; p.(G928S)	C3U	?		/
**32**	F	52	0.52	0.44 (53)	2	2	0	0	0	4	c.3389C>T; p.(T1130M)	C5	c.3907G>C; p.(A1303P)	C5	M+M
**33**	M	64	0.53	0.48 (65)	0	2	0	0	0	2	c.3421C>T; p.(R1141*)	C5	c.1108A>G; p.(N370D)	C3LB	N+M
**34**	M	31	0.49	0.46 (32)	2	2	0	0	0	4	c.3421C>T; p.(R1141*)	C5	c.3421C>T; p.(R1141*)	C5	N+N
**35**	F	53	0.40		3	3	0	0	0	6	c.3421C>T; p.(R1141*)	C5	c.3421C>T; p.(R1141*)	C5	N+N
**36**	M	61	0.42	0.51 (62)	0	3	3	2	0	8	c.2252T>A; p.(M751K)	C5	c.3907G>C; p.(A1303P)	C5	M+M
**37**	F	30	0.54		3	3	0	0	0	6	c.3669G>A; p.(W1223*)	C5	c.3669G>A; p.(W1223*)	C5	N+N
**38**	M	11	0.38		1	2	0	0	0	3	c.3940C>T; p.(R1314W)	C5	c.3887G>A; p.(G1296D)	C5	M+M
**39**	M	10	0.56		1	0	0	0	0	1	c.3940C>T; p.(R1314W)	C5	c.3887G>A; p.(G1296D)	C5	M+M

**40**	M	39	0.71		0	2	0	0	0	2	c.3940C>T; p.(R1314W)	C5	?		/
**41**	F	21	0.48		2	2	0	0	0	4	c.3421C>T; p.(R1141*)	C5	c.1171A>G; p.(R391G)	C3LP	N+M
**42**	F	55	0.37		3	3	0	1	0	7	c.2787+1G>T; p.(G972=)	C5	c.2787+1G>T; p.(G972=)	C5	D+D
**43**	F	44	0.42		2	3	0	0	0	5	c.1552C>T; p.(R518*)	C5	c.3662G>A; p.(R1221H)	C5	N+M
**44**	F	30	0.36		2	2	0	0	0	4	c.3421C>T; p.(R1141*)	C5	c.2432C>T; p.(T811M)	C5	N+M
**45**	M	54	0.67		0	2	1	0	0	3	c.3902C>T; p.(T1301I)	C4	?		/
**46**	M	20	0.64		2	2	0	0	0	4	c.3421C>T; p.(R1141*)	C5	c.3941G>A; p.(R1314Q)	C5	N+M
**47**	F	66	0.59		2	4	0	0	0	6	c.3775delT; p.(Y1259Mfs*33)	C5	c.3775delT; p.(Y1259Mfs*33)	C5	D+D
**48**	M	29	0.25	0.25 (30)	0	2	0	0	0	2	c.3421C>T; p.(R1141*)	C5	c.3421C>T; p.(R1141*)	C5	N+N
**49**	M	20	0.39		2	2	0	0	0	4	c.1553G>A; p.(R518Q)	C5	c.1553G>A; p.(R518Q)	C5	M+M
**50**	F	46	0.39		1	2	0	0	0	3	c.3421C>T; p.(R1141*)	C5	c.3437T>C; p.(F1146S)	C3U	N+M
**51**	F	52	0.61		2	3	0	0	0	5	c.1944-1G>C; p.(C648=)	C5	c.3907G>C; p.(A1303P)	C5	D+M
**52**	M	49	0.53		2	3	0	0	0	5	c.1944-1G>C; p.(C648=)	C5	c.3907G>C; p.(A1303P)	C5	D+M
**53**	F	39	0.55		1	2	0	0	0	3	c.2304C>A; p.(Y768*)	C5	c.3421C>T; p.(R1141*)	C5	N+N
**54**	M	43	0.54		3	2	0	0	0	5	c.3941G>A; p.(R1314Q)	C5	c.1171A>G; p.(R391G)	C3LP	M+M
**55**	M	40	0.43		2	3	0	0	0	5	c.1553G>A; p.(R518Q)	C5	c.1553G>A; p.(R518Q)	C5	M+M

**56**	F	80	0.88	1	4	0	0	0	5	c.2279G>A; p.(R760Q)	C5	c.3421C>T; p.(R1141*)	C5	N+M
**57**	F	46	0.62	2	2	0	0	0	4	c.1553G>A; p.(R518Q)	C5	c.1553G>A; p.(R518Q)	C5	M+M
**58**	M	59	0.45	2	4	1	0	0	7	c.3412C>T; p.(R1138W)	C5	c.3421C>T; p.(R1141*)	C5	N+M
**59**	F	40	0.49	1	2	0	0	0	3	c.4182delG; p.(K1394Nfs*8)	C5	c.3787G>A; p.(G1263R)	C4	D+M
**60**	F	30	0.51	2	2	0	0	0	4	c.3421C>T; p.(R1141*)	C5	c.3907G>C; p(.A1303P)	C5	N+M
**61**	F	48	0.47	3	2	1	0	0	6	g.1664_72917del	C5	c.4153G>C; p.(A1358P)	C4	D+M
**62**	M	33	0.08	3	2	0	0	0	5	c.3507-3C>A	C3U	c.2831C>T; p.(T944I)	C3U	D+M
**63**	M	30	0.25	0	2	0	0	0	2	c.3421C>T; p.(R1141*)	C5	c.3421C>T; p.(R1141*)	C5	N+N
**64**	F	52	0.26	1	1	0	0	0	2	c.4153G>C; p.(A1385P)	C4	Del24-27	C5	D+M
**65**	F	42	0.30	3	2	1	0	0	6	c.3421C>T; p.(R1141*)	C5	c.3421C>T; p.(R1141*)	C5	N+N
**66**	M	53	0.52	0	2	0	0	0	2	c.3421C>T; p.(R1141*)	C5	c.3421C>T; p.(R1141*)	C5	N+N
**67**	F	57	0.36	0	2	0	0	0	2	c.3421C>T; p.(R1141*)	C5	c.4375C>T; p.(R1459C)	C4	N+M
**68**	F	25	0.23	1	2	0	0	0	3	c.2420G>A; p.(R807Q)	C5	c.2787+1G>T; p.(G972=)	C5	D+M
**69**	F	59	0.61	3	2	2	0	0	7	c. 3437T>C; p.(F1146S)	C3U	c.4105G>A; p.(E1369K)	C4	M+M
**70**	M	14	0.38	2	2	0	0	0	4	c.3871G>A; p.(A1291T)	C5	c.3887G>A; p.(G1296D)	C5	M+M
**71**	F	24	0.40	1	2	0	0	0	3	c.1171A>G; p.(R391G)	C3LP	c.3421C>T; p.(R1141*)	C5	N+M

**72**	F	54	0,63	2	2	1	1	1	7	c.651G>A; p.(W217*)	C5	c.1553G>A; p.(R518Q)	C5	N+M
**73**	F	30	0.62	2	2	0	0	0	4	c.3364delT; p.(S1122Lfs*33)	C5	c.3364delT; p.(S1122Lfs*33)	C5	D+D
**74**	M	49	0.48	2	2	1	0	0	5	c.3421C>T; p.(R1141*)	C5	c.3421C>T p.(R1141*)	C5	N+N
**75**	M	53	0.18	2	3	1	0	0	6	c.3421C>T; p.(R1141*)	C5	c.3421C>T; p.(R1141*)	C5	N+N
**76**	F	52	0.97	2	3	1	0	0	6	c.2359G>A; p.(V787I)	C4	c.196dup; p.(S66Ffs*35)	C4	M+M
**77**	F	53	0.63	3	3	1	0	0	7	c.4321C>T; p.(R1141*)	C5	c.1943+2T>C	C3LP	N+M
**78**	F	18	0,50	2	1	0	0	0	3	c.1552C>T; p.(R518*)	C5	c.3421C>T; p.(R1141*)	C5	N+N

M= male; F= female. Age (years) at first PPi measurement. Any additional sample is displayed to the right in the same column with the age at sampling between parentheses. S= skin, E= eyes, V= vascular, C= cardiac, G= gastrointestinal, T= total cumulative Phenodex score. Phenodex score was re-evaluated at each sampling but did not change in any patient. *ABCC6* variants: C3 are variants of unknown significance and pathogenicity was estimated as either unknown (U), likely pathogenic (LP) or likely benign (LB). C4 are likely pathogenic and C5 are pathogenic variants. Genotype categories are shown in the Cat. tab as combinations of D=deletions/frameshifts/splice site, M=missense, N=Nonsense.

Supplementary Table S3. Characteristics of the heterozygous carrier and control study cohort.

HETEROZYGOUS CARRIERS						
	**Sex**	**Age**	**PPi**	**Variant 1 & Class**		**Cat.**
**1**	M	29	0.48	c.*3421C>T (p.R1141*)	C5	N
**2**	M	30	0.85	c.*3421C>T (p.R1141*)	C5	N
**3**	M	33	0.92	c.*3421C>T (p.R1141*)	C5	N
**4**	F	59	0.83	c.3941G>A (p.R1314Q)	C5	M
**5**	F	53	0.84	c.3941G>A (p.R1314Q)	C5	M
**6**	M	54	0.72	c.*3421C>T (p.R1141*)	C5	N
**7**	M	46	0.91	c.*3421C>T (p.R1141*)	C5	N
**8**	F	45	0.63	c.*3421C>T (p.R1141*)	C5	N
**9**	M	29	0.91	c.3389C>T (p.T1130M)	C5	M

**10**	F	28	0.82	c.1171A>G (p.R391G)	C3LP	M
**11**	M	33	0.83	c.*3421C>T (p.R1141*)	C5	N
**12**	F	61	0.87	c.*3421C>T (p.R1141*)	C5	N
**13**	M	62	0.83	c.3941G>A (p.R1314Q)	C5	M
**14**	F	42	0.70	c.3887G>A (p.G1296D)	C5	M
**15**	M	58	1.14	c.1171A>G (p.R391G)	C3LP	M
**16**	M	61	0.63	c.2252T>A (p.M751K)	C5	M
**17**	F	35	0.59	c.2252T>A (p.M751K)	C5	M
**18**	M	69	0.59	c.3907G>C (p.A1303P)	C5	M
**19**	F	64	0.85	g.*65280_78907del	C5	D
**20**	M	62	0.63	c.3907G>C (p.A1303P)	C5	M
**21**	F	24	0.58	c.3907G>C (p.A1303P)	C5	M
**22**	F	64	0.83	c.1171A>G (p.R391G)	C3LP	M
**23**	M	54	0.35	c.3902C>T (p.T1301I)	C4	M
**24**	M	56	0.98	c.3941G>A(p.R1314Q)	C5	M
**25**	M	40	1.53	c.1171A>G (p.R391G)	C3LP	M
**26**	F	37	0.32	c.3736-1G>A(p.?)	C4	D
**27**	M	63	1.12	c.3447del (p.N1150Tfs*5)	C5	N
**28**	M	39	0.41	c.1171A>G (p.R391G)	C3LP	M
**29**	M	20	0.38	c.2787+1G>T	C5	D
**30**	M	67	0.50	c.3907G>C (p.A1303P)	C5	M
**31**	F	34	0.75	c.3907G>C (p.A1303P)	C5	M
**32**	F	43	0.74	c.3775del (p.W1259Gfs*14)	C5	N
**33**	F	52	0.67	c.3979G>A (p.G1327R)	C4	M
**34**	M	48	0.95	c.3941G>A (p.A1314Q)	C5	M
**35**	F	35	0.51	c.3421C>T (p.R1141*)	C5	N
**36**	F	50	0.80	c.3289C>A (p.L1097I)	C3U	M
**37**	M	51	0.47	c.3941G>A (p.R1314Q)	C5	M
**38**	F	33	0.62	c.3421C>T (p.R1141*)	C5	N
**39**	F	42	0.31	c.3421C>T (p.R1141*)	C5	N
**40**	M	45	0.54	c.196dup (p.S66Ffs*35)	C4	D
**41**	M	30	0.66	c.C4182del (p.4182del)	C5	D
**42**	M	48	0.65	c.1171A>G (p.R391G)	C3LP	M
**43**	F	40	0.99	c.1955C>T (p.T652M)	C3U	M

**44**	M	32	0.74	c.1171A>G (p.R391G)	C3LP	M
**45**	F	60	0.92	c.1171A>G (p.R391G)	C3LP	M
**46**	M	61	1.01	c.3161C>T (p.T1054M)	C3U	M
**47**	M	38	0.68	c.2252T>A (p.M751K)	C5	M
**48**	M	25	0.47	c.1171A>G (p.R391G)	C3LP	M
**49**	M	37	0.64	c.3907G>C (p.A1303P)	C5	M
**50**	M	29	0.84	c.1171A>G (p.R391G)	C3LP	M
**51**	M	39	0.66	c.2420G>A (p.R807Q)	C5	M
**52**	F	12	0.74	c.3421C>T (p.R1141*)	C5	N
**53**	F	42	0.46	c.3421C>T (p.R1141*)	C5	N
**54**	M	44	0.70	c.4477T>C (p.F1493L)	C3U	M
**55**	F	38	0.95	c.3421C>T (p.R1141*)	C5	N
**56**	F	52	0.61	c.3941G>A (p.A1314Q)	C5	M
**57**	F	26	0.58	c.3907G>C (p.A1303P)	C5	M
**58**	F	33	0.44	c.3421C>T (p.R1141*)	C5	N
**59**	M	35	0.58	c.1171A>G (p.R391G)	C3LP	M
**60**	F	52	0.84	c.2359G>A (p.V787I)	C4	M
**61**	M	18	0.43	c.3447del (p.N1150Tfs*5)	C5	D
**62**	F	37	0.76	c.3161C>T (p.T1054M)	C3U	M
**63**	M	33	0.43	c.3421C>T (p.R1141*)	C5	N
**64**	F	31	0.51	c.1873G>A (p.G625R)	C3U	M
**65**	F	69	0.72	c.2635G>A (p.A879T)	C3U	M
**66**	F	68	0.83	c.4104del (p.D1368Efs*35)	C5	D
**67**	M	64	0.77	c.3979G>A (p.G1327R)	C4	M
**68**	F	28	0.91	c.3421C>T (p.R1141*)	C5	N
**69**	M	58	1.13	c.1171A>G (p.R391G)	C3LP	M

CONTROLS

	**Sex**	**Age**	**PPi**
**1**	M	27	0.97
**2**	M	53	1.28
**3**	F	40	0.98

**4**	F	32	1.06
**5**	F	50	0.95
**6**	F	28	1.39
**7**	F	50	0.76
**8**	M	24	0.64
**9**	F	29	1.11
**10**	M	33	0.82
**11**	F	43	0.99
**12**	M	49	0.81
**13**	M	42	1.13
**14**	M	41	0.90

Sex is M (male) or F (female), age is shown in years and PPi values are in μM. The left hand column of PPi shows the value corresponding to the age. ABCC6 variants are shown with their Sherloc classification. C3LP are variants of unknown significance but likely pathogenic. C4 and C5 variants are (likely) pathogenic. Genotype categories – akin to the PXE cohort – are shown in the Cat. tab: D= deletions/frameshifts/splice site, M= missense, N= nonsense.
